# Micro-Scale Particle Tracking: From Conventional to Data-Driven Methods

**DOI:** 10.3390/mi15050629

**Published:** 2024-05-08

**Authors:** Haoyu Wang, Liu Hong, Leonardo P. Chamorro

**Affiliations:** 1 Department of Mechanical Science and Engineering, University of Illinois Urbana-Champaign, Urbana, IL 61801, USA; haoyu17@illinois.edu (H.W.); liuhong2@illinois.edu (L.H.); 2 Department of Aerospace Engineering, University of Illinois Urbana-Champaign, Urbana, IL 61801, USA; 3 Department of Civil and Environmental Engineering, University of Illinois Urbana-Champaign, Urbana, IL 61801, USA; 4 Department of Geology, University of Illinois Urbana-Champaign, Urbana, IL 61801, USA

**Keywords:** micro-scale positioning, particle tracking velocimetry, fluid mechanics, data-driven method, deep learning, neural networks

## Abstract

Micro-scale positioning techniques have become essential in numerous engineering systems. In the field of fluid mechanics, particle tracking velocimetry (PTV) stands out as a key method for tracking individual particles and reconstructing flow fields. Here, we present an overview of the micro-scale particle tracking methodologies that are predominantly employed for particle detection and flow field reconstruction. It covers various methods, including conventional and data-driven techniques. The advanced techniques, which combine developments in microscopy, photography, image processing, computer vision, and artificial intelligence, are making significant strides and will greatly benefit a wide range of scientific and engineering fields.

## 1. Introduction

In the rapidly evolving landscape of technology, the demand for precise and efficient positioning techniques has become increasingly paramount, especially in the realm of micro-scale applications. Micro-scale positioning involves the precise identification and tracking of particles, objects, or devices at a small scale, often ranging from one hundred nanometers to a few millimeters. This specialized field is integral to numerous cutting-edge technologies, including micro-electromechanical systems (MEMS), biomedical devices, and miniature sensors. The development and refinement of micro-scale positioning techniques have far-reaching implications, offering solutions to challenges in fields such as healthcare, robotics, and telecommunications.

At the center of positioning techniques are the locating tasks, which are aimed to obtain the accurate location information of the interest targets, such as cells, tracers, droplets, and particles. Characterizing the single and collective motion of tracer particles is essential for reconstructing flow fields and their properties. Particle tracking velocimetry (PTV), a powerful non-intrusive technique that uses a Lagrangian approach by tracking individual particles in consecutive images with sufficiently short time separation, is introduced to tackle this task. This article does not cover other image-based techniques like particle image velocimetry (PIV), which use the Eulerian approach. In PTV, accuracy is typically defined as how closely the reconstructed particle locations, trajectories, or flow properties match the ground truth. This is measured using quantitative metrics such as root-mean-square (RMS) error, mean squared error (MSE), and signal-to-noise ratio (SNR), which evaluate deviations in pixel values, particle displacements, and flow statistics.

Over the last few decades, the development of particle tracking velocimetry (PTV) has evolved into multiple types, including scanning PTV [1,2], defocusing PTV [3], tomographic PTV [4,5,6,7,8], and various other variants [9,10,11,12,13]. Distinct developments include single-view [14] and multi-view strategies, such as stereo-PTV [15,16,17,18]. In addition to advancements in measurement, particle linking has been explored to reduce computational time and increase particle density. Inspired by the tomographic method [4] and iterative particle reconstruction [19], ‘Shake-The-Box (STB)’ [20,21] has been successful in detecting and monitoring individual particles under high particle image densities, with particle counts ranging from tens to hundreds of thousands per time interval. Noteworthy progress in micro-scale PTV (μPTV) includes confocal microscopy [22,23,24,25], defocusing-based approaches [26,27,28], stereomicroscopy [29,30], and synthetic aperture refocusing methods [31,32]. In addition, significant advancements have been made using single cameras with the plenoptic method (also called light field) [33,34,35].

**Figure 1:**
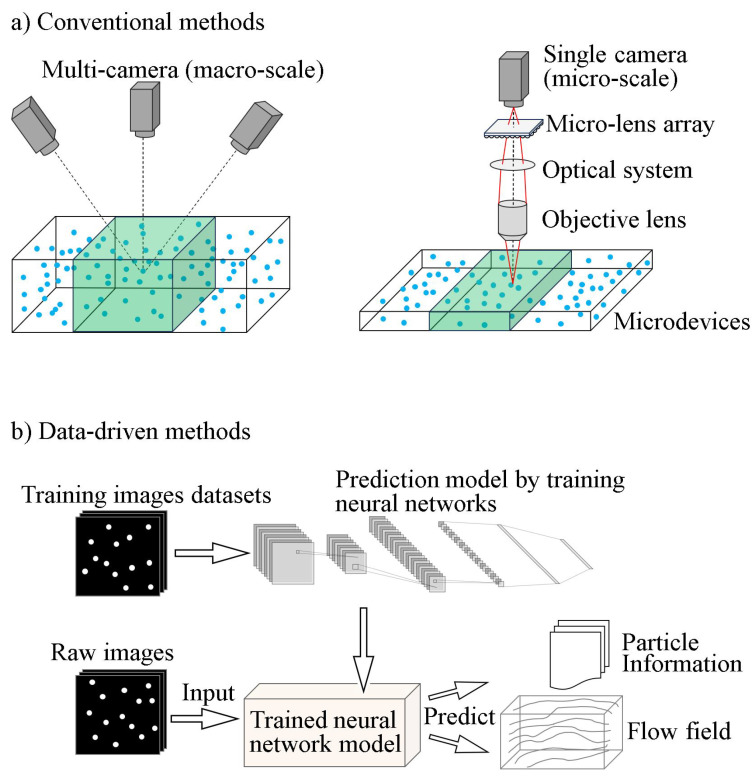
(**a**) General schematics of conventional macro-scale and micro-scale PTV (not showing reconstruction process). (**b**) A typical pipeline of data-driven PTV methods.

Traditional PTV methods have laid a solid foundation for Lagrangian description by tracking individual particles through a flow field. Building on this foundation, recent advances have integrated cutting-edge artificial intelligence technologies, significantly expanding the capabilities and applications of PTV. The advent of data-driven PTV harnesses the power of machine learning to enhance analysis and interpretation. Examples of such advancements include PTV using shallow neural networks [36], DeepPTV [37], PINN-augmented PTV [38], LSTM-enhanced PTV [39], and stochastic particle advection velocimetry (SPAV) [40], among others. Each of these approaches offers unique advantages in terms of accuracy, processing speed, and the ability to handle complex flow scenarios. Hybrid methodologies that combine conventional tracking with AI techniques represent a significant leap forward, opening new avenues for research and application in particle dynamics. A generic illustration of both conventional and data-driven methods of PTV is shown in Figure 1.

This article aims to provide an overview of various particle tracking techniques complemented with flow reconstruction methods, both conventional and data-driven, particularly applicable at the micro-scale. The general features of PTV across a variety of scales are discussed, given their applicability at the micro-scale.

## 2. Conventional Micro-Scale Particle Tracking

### 2.1. Basics for Micro-Scale Particle Tracking Velocimetry (μPTV)

The typical 3D particle tracking process, as outlined in Schröder and Schanz [41], involves capturing images simultaneously from single or multiple camera (or view) perspectives. The identification of pixels that display peak intensity values occurs within each image on every camera. The extension of lines-of-sight (LOS) for these peak pixels into the targeted volume is accomplished through the application of 3D camera calibration. The accurate determination of a particle’s 3D position relies on the intersection of LOS from identified particle image peaks across different cameras, maintaining an acceptable triangulation error threshold of approximately 1 pixel. The 3D positions of particles are stored in a list, instead of being represented in voxel intensity distributions. Subsequent stages include the reconstruction of sets of 3D particle positions for each time step by incorporating all detected peaks across cameras. A tracking algorithm is then deployed to ensure consistent identification of the same imaged particle throughout the corresponding timeline of 3D particle reconstructions. This tracking process serves to systematically construct extended and detailed 3D particle trajectories. More detailed information on μPTV principles can be found in, e.g., Ponchaut et al. [42] and Dabiri and Pecora [43].

### 2.2. 3D Particle Identification and Reconstruction: Hardware and Algorithms

Conventional PTV technology has seen significant development in hardware and software. We briefly highlight several milestone breakthroughs on 3D particle tracking applicable to micro-scale measurements.

#### 2.2.1. Holographic Particle Tracking

A single camera holography is based on the interference between light scattered from objects and reference light to encode depth information into holograms. Progress in high-power laser technology and distinct off-axis reference wave accelerated this development. Standard practices involve the use of photo-refractive and other nonlinear optics materials to record holograms.

The use of advanced image processing and decoding methods, such as encryption, pattern recognition, associative memory, and neural networks [44], facilitates the feasibility of real-time holographic recording and reconstruction [45,46]. Through the fusion of diffractive holography and digital encoding/decoding methods, a digital holographic microscope (DHM) has been developed. It is also employed for measuring 3D velocity fields and 3D particle tracking. In DHM, the holographic images of tracer particles in a flow are captured directly using a digital image recording device. Furthermore, to overcome the comparatively low spatial resolution induced by pixel size limitations, in-line DHM was developed by using in-line digital holography [47]. As a result, the DHM-PTV technique is a robust method to measure 3C-3D velocity field information of a microscale flow with a reasonable spatial resolution [48].

#### 2.2.2. Confocal Microscopy

**Figure 2:**
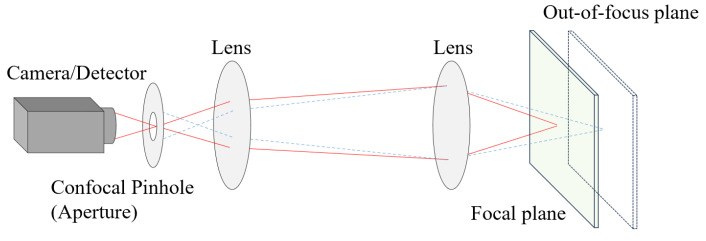
Schematics of the generic working principle of a confocal microscope. Adapted from Semwogerere and Weeks [50].

Confocal microscopy (Figure 2) employs selective exclusion of light outside the microscope’s focal plane to produce sharp images of a specimen, reducing haze and improving contrast compared to conventional microscopy. This technique captures multiple cross sections of investigation volume, allowing for better observation of fine details. Finally, by assembling a series of thin slices along the vertical axis, confocal microscopy enables the construction of three-dimensional reconstructions of the target [49,50].

Confocal microscopy has also been applied to particle tracking [51,52,53]. One representative example is the confocal laser scanning microscopy (CLSM) [54,55]. The basic principle of CLSM involves the point-scanning of the laser excitation and the spatially filtered fluorescence signal emitted from the focal point onto the confocal point. Compared to conventional microscopy, CLSM offers the advantage of providing clear images for identifying particle positions within each slice or focal plane. This is due to the ability of the confocal microscope to exclude out-of-focus light, resulting in improved image clarity and enhanced visualization of specimen details. However, image degradation near the edges of microtubes is a potential issue due to increased lens effects and internal reflections, particularly in regions with significant velocity changes.

The effectiveness of optical slicing in CLSM may diminish with lower magnification and numerical aperture objectives. Challenges may also arise in compensating for refractive index mismatches and correcting curved image planes, especially in cases with thicker and more curved microtube walls. The velocity of tracer particles in CLSM is constrained by the scanning speed, given its reliance on the scanning frequency. The scanning speed of CLSM is also limited by the galvanometric steering no more than 1 fps, requiring innovative designs to enhance scanning rates for future microfluidic applications with higher velocity ranges. Furthermore, the relatively low exposure time for each slice requires very high-power illumination, which may not be suitable for live microorganism tracer tracking, as it could potentially harm the organisms under observation.

#### 2.2.3. Structured Illumination Microscopy

When conducting micro-scale particle tracking through microscopy, the spatial resolution of particle reconstruction may be influenced by signals from tracer particles outside the focal plane, particularly in the case of relatively thick microchannels. The presence of a strong background signal is a significant bottleneck for 3D micro-particle tracking methods based on volumetric illumination. Structured illumination microscopy particle tracking velocimetry (SIM-PTV) has the capability to eliminate a substantial portion of the background signal by employing non-zero spatial frequency illumination. This approach prioritizes the illumination of in-focus particles by combining two such images captured by a double-exposure SIM. The standard deviations in the SIM-PTV are roughly 55%, which is lower compared to the volumetric illumination PTV. The velocity error in SIM-PTV is about 5%, contrasting with roughly 20% in volumetric illumination PTV [56,57].

#### 2.2.4. Tomographic PTV

**Figure 3:**
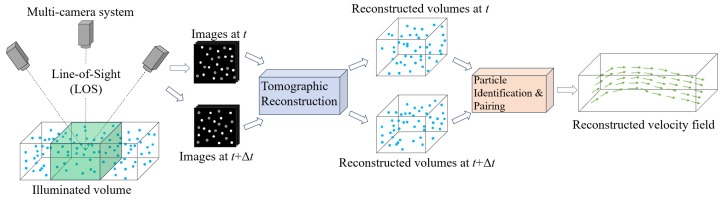
Typical procedure of tomographic PTV. Adapted from Elsinga et al. [4].

Elsinga et al. [4] presented a tomographic technique (see Figure 3), which is designed to provide instantaneous 3D velocity field measurements, making it suitable for analyzing flows in various regimes and irrespective of flow speed. The tomographic approach operates on several core principles, revolving around the illumination, recording, and reconstruction of tracer particles within a three-dimensional measurement volume. This technique relies on optical tomography and simultaneous views of illuminated particles and their subsequent 3D reconstruction to form a light intensity distribution in a multi-camera system. The original reconstruction process results in a discretized 3D array of light intensity across voxels by employing the multiplicative algebraic reconstruction technique (MART) algorithm [58], which is an iterative approach used in medical imaging to reconstruct high-quality images from projection data by iteratively updating an initial image estimate using a multiplicative correction factor. Tomographic PIV uses direct cross-correlation to calculate the velocity vector field; however, in tomographic PTV, 3D particle locations need to be captured and utilized through the identification and pairing process [6]. The identified 3D positions of particles are sequentially connected frame by frame to deliver instantaneous 3D velocity field measurements and particle trajectories. The tomographic system is versatile, capable of analyzing flows across various regimes and regardless of flow speeds.

Despite its capabilities, tomographic measurement does have limitations, particularly with regard to the complexity of the reconstruction process and the sensitivity to the setting parameters [4]. One key challenge arises from the fact that a single set of projections can correspond to multiple potential 3D objects, making it difficult to ascertain the most probable 3D particle distribution from the reconstructions. In addition, the accuracy of the reconstruction is influenced by various factors, such as the number of viewing directions, particle seeding density, the precision of the calibration (preferably within approximately 0.4 pixels), and the presence of image artifacts [4]. These factors collectively affect the reliability and precision of the reconstructed data, highlighting important considerations for the implementation and interpretation of tomographic PTV measurements.

#### 2.2.5. Iterative Particle Reconstruction (IPR)

Wieneke [19] proposed ’iterative particle reconstruction’ (IPR), which aims to track the motion of illuminated particles in space and time by comparing images with projections calculated from the particle distribution in the volume. Unlike voxel representation techniques like MART, IPR updates both particle position and particle intensity. The algorithm iteratively updates and corrects the particle distribution in the volume, offering advantages over single-pass techniques (MLOS [59] and 3D PTV). IPR has shown better performance for seeding densities up to 0.05 ppp, with lower particle position error compared to the MART technique. Also, IPR shows potential for dealing with non-uniform imaging conditions, incorporating locally varying optical transfer functions (OTFs, a variant of Fourier transform used in imaging systems to analyze spatial frequencies) to improve results. The algorithm shows promise for advanced time-series analysis with Lagrangian particle tracking, offering potential for improved particle reconstruction using time-coherence constraints.

However, IPR faces various challenges, being the most critical its tendency to deviate strongly from the true particle distribution at sufficiently high seeding densities [19]. It may also result in the selection of fewer particles and higher particle intensity, consequently increasing the occurrence of ghost particles. The convergence of IPR is challenged above 0.05 ppp when multiple 3D distributions of particles are consistent with the recorded images. This limitation affects the reliability of the method for higher seeding densities. Furthermore, the method’s performance may be affected by non-uniform imaging conditions, such as astigmatism or defocusing effects, which can compromise the sharpness of particle images in certain regions of the domain.

#### 2.2.6. A 4D PTV Approach: ‘Shake-The-Box’ (STB)

The classical tomographic PIV/PTV has limitations such as the measurement error of velocity vectors introduced by ghost particles, the particle location error brought by the discretized voxel space, and the demanding requirement on computational and memory resources [21]. The 3D PTV with IPR [19] improves the working threshold of particle image density to 0.05 ppp by ‘shaking’ the particle to reach a local minimum residual. However, both methods still cannot eliminate the influence caused by ghost particles, (spurious particles or image artifacts that can corrupt the velocity measurements) because of their overlooking on the prior knowledge of previously analyzed image data.

Schanz et al. [21] introduced the so-called ‘Shake-The-Box’ (STB) that uses the inter-correlation of temporal and spatial information and leverages extrapolation of known particle trajectories to better estimate the particle distribution. The typical tracking-reconstruction process is reversed into a prediction-identification sequence. The anticipated particle distribution serves as an initial input for the IPR process. This process initially rectifies prediction errors and subsequently identifies new particles not currently tracked. The outcome is an efficient method for swiftly handling 3D data with elevated particle concentrations. This method effectively captures the actual particles and minimizes the generation of ghost counterparts [21].

There have been many advancements based on the STB concept. Recently, Tan et al. [60] shared an open-source OpenLPT package, which successfully reduced the ghost particles by roughly 80% at 0.125 ppp. Jahn et al. [61] revised the IPR by increasing the particle image density threshold by 3 times particle image densities, and improved speed, accuracy, and robustness against image noise while maintaining low computational costs. A new Two-Pulse Shake-The-Box (TP-STB) scheme was introduced by Novara et al. [62]. An application of this approach in a micro flow through porous media was described in Bagheri and Mirbod [63].

#### 2.2.7. Particle Tracking with Plenoptic Imaging

The plenoptic (light field) camera is built upon the concept of plenoptic functions, which parameterize the light field into a 5D function that encodes the position and direction of each light ray. Therefore, a single camera can capture the 3D information of objects with a plenoptic encoding format [64]. In general, plenoptic imaging requires a microlens array (MLA) mounted closely to the camera sensor (with a distance of the MLA focal length). The MLA is typically a square glass substrate covered by N × N (N is usually hundreds) small polymer lenses of 100 to 1000 μm in radius.

Lynch et al. [33] first applied a single light field camera for 3D-3C particle image velocimetry (LF-PIV). Fahringer et al. [34] upgraded the previous work by adopting the multiplicative algebraic reconstruction technique (MART) [58]. However, these methods rely on iterative calculations to optimize, which consume a significant amount of computational time (typically on the order of 1/2 h) and resources [34], which is not efficient considering the typical amount of images produced in an experiment.

**Figure 4:**
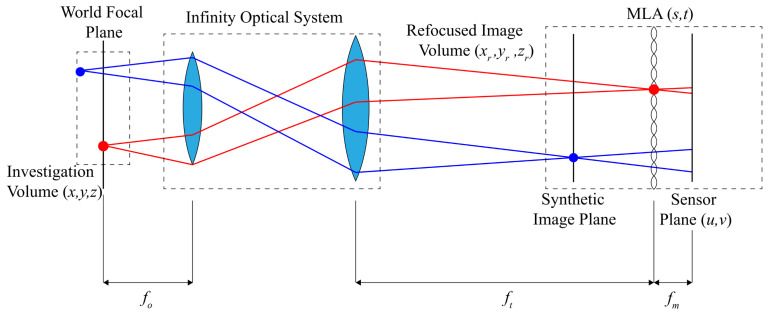
Illustration of the experimental setup of LF-μPIV/PTV. Adapted from Hong and Chamorro [35].

Hong and Chamorro [35] developed a GPU-accelerated non-iterative algorithm for LF-μPIV/PTV integrating ray-tracing, kd-tree search, cloud point classification, and density-based spatial clustering of applications with noise (DBSCAN) and reducing the processing time under 100 ms. The non-iterative method recovers 3D rays based on pixel coordinates on the sensor plane and corresponding lenslet center position on the MLA plane, as shown in the schematics of Figure 4. Then, by reconstructing the light rays with the intersection information and elongating these rays into the measurement volume, the intersection points (voxels) where the real particles are most likely to be are determined. However, the 3D reconstruction on particles located near the focal plane and high velocity flow field near the center of the camera view slightly depart from the ground truth. The reconstruction accuracy may also be affected if the MLA plane is too close to the camera sensor, leading to the suggestion of using MLA with shorter focal length at the cost of depth of view. Later, Gu et al. [65] considered the diffraction issue neglected by geometrical optics in microscopes and improved the reconstruction efficiency with a low-rank decomposition-based deconvolution (LRDD) method. They also proposed a cross-validation matching (CVM) algorithm [66] to improve the performance of LF-μPIV/PTV under high particle image density conditions.

### 2.3. Key Takeaways from Conventional Methods

Conventional techniques for particle tracking and flow reconstruction have proven highly applicable across various scenarios. Depending on specific working conditions, different techniques are favored; see Table 1. Tomographic-based approaches and its variants remain prevalent for large-scale tracking tasks. Under high seeding densities, STB is commonly employed. At micro-scale with single-camera setups, due to space constraints, LF-μPIV/PTV is preferred. Confocal microscopy and structured illumination techniques are adopted when spatial resolution and denoising are critical. In some cases, further denoising and distortion/aberration correction procedures [67,68,69,70,71,72,73,74,75,76] are adopted to augment the data and obtain more reliable results.

Standard methods for particle tracking have demonstrated versatility and adaptability to diverse experimental setups. There are various avenues for improvements. For example, advancements in illumination instrument and imaging technology could enhance spatial resolution and reduce noise, enabling more accurate and detailed particle tracking in challenging environments. Improvements in computational algorithms and processing techniques could optimize particle identification, tracking, and 3D reconstruction, particularly in scenarios with high particle densities, limited viewing spaces, or complex flow patterns. Among these, the integration of machine learning and artificial intelligence approaches (i.e., data-driven methods) could further enhance the overall efficiency and accuracy of particle tracking algorithms by automating manual processes and enabling real-time tracking in dynamic environments. This is discussed next.

## 3. Artificial Intelligence Augmented Particle Tracking

### 3.1. Data-Driven Particle Tracking and Associated Flow Reconstruction

The amount of image data produced by PTV makes it highly suitable to adopt data-driven approaches to upgrade or replace conventional particle tracking and flow reconstruction algorithms. Base work [77,78,79] leveraged multilayer shallow neural networks (SNNs) to fix particle matching issues. Recent efforts also involve SNNs to improve the overall quality of flow reconstruction [36,38]. Recent breakthroughs in deep learning and convolutional neural networks (CNNs) has showed their transferability from traditional computer vision tasks to PIV/PTV applications. This synergy between data-driven approaches and PTV presents a significant advantage, particularly in micro-scale particle tracking.

Newby et al. [80] proposed a three-layer CNN to automatically locate submicro-scale particles from 2D/3D videos. Liang et al. [81] proposed a novel graph neural network model called GotFlow3D to estimate fluid motion from consecutive particle images. Dreisbach et al. [82] improved the successful rate of particle detection in defocusing PTV, even for overlapped particles and under aberration and uneven illumination conditions. Sax et al. [83] investigated a new hybrid method to improve existing defocusing PTV techniques, which combines Faster R-CNN [84] with a simple maximum intensity detection algorithm, outperforming both neural networks and traditional detection algorithms. Franchini and Krevor [85] improved 3D particle localization in astigmatic optical systems using an LSTM-CNN model, achieving high accuracy and robustness, especially for overlapped and low-intensity particles, at an overall accuracy above 90%. Liang et al. [86] proposed a two-stage double-CNN-involved pipeline to segment and identify particles on the sub-pixel level. For flow visualization and velocity field reconstruction, there are some emerging methods: reconstructing 2D time-varying flow fields with particle tracing and Lagrangian representations [87,88], using deep learning to reconstruct vector fields from streamlines [89,90], and with no particles involved but purely applying a hybrid neural network (HyFluid) to process sparse multi-view videos and infer fluid density and velocity fields [91,92,93]. Other than neural network models, transformer models [94,95] are also becoming widely used in the domain of complex flow prediction. Another novel method called Kernelized Lagrangian particle tracking (KLPT) [96] integrates the kernel trick with data assimilation to obtain the optimal mapping function.

The integration of data-driven methods and particle tracking with flow reconstruction aims to address the inherent challenges faced by conventional techniques, offering promising solutions in fluid dynamics research. Here, we illustrate various representative learning regimes and examples on how data-driven methodologies have augmented the micro-scale particle tracking and flow reconstruction, extending them into a broader application regime.

### 3.2. Shallow Neural Networks-Assisted Particle Tracking

A shallow neural network (SNN) is characterized by its relatively small number of layers, making its structure simpler and more concise compared to deep neural networks (DNNs). Despite its simple architecture, SNNs have proven to be remarkably effective in certain tasks, as demonstrated in, e.g., Gim et al. [36] and Wang et al. [38], who successfully reconstructed three-dimensional particle positions with high accuracy. This efficiency stems from the reduced computational complexity of SNNs, allowing for faster processing times without sacrificing performance. By leveraging fewer weights and layers, SNNs offer a streamlined approach to neural network modeling, particularly advantageous in scenarios where computational resources are limited or where rapid inference is likely crucial.

#### 3.2.1. Real-Time 3D Particle Tracking with a Two-Layer Neural Network

**Figure 5:**
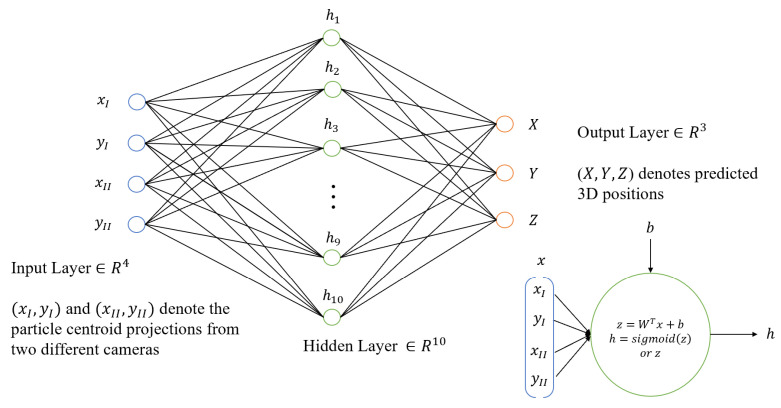
The structure of the suggested two-layer shallow neural network. The first layer uses sigmoid as the activation function; the second layer only has linear mapping. Adapted from Gim et al. [36].

Gim et al. [36] demonstrated that using a two-layer shallow neural network (see schematics of Figure 5) can significantly drop the computational load in obtaining the mapping functions but still reach high accuracy for stereoscopic 3D particle tracking. They used simulated particles, positioned randomly or flowing, to record their 3D locations as the ground truth data for training. The numerical evaluation on the reconstruction algorithm across different mesh resolutions showed that the errors were reasonable. Also, they validated the SNN algorithm by measuring the Marangoni mixing and bursting flow of a mixture droplet (water and 50 wt% ethanol) during evaporation. A stereo-camera system was built to record the ground truth images. The reconstruction on 1000 particles took around 0.5 s, with 99.9% of particles successfully matched in their positions; the reconstruction time on the velocity field was approximately a few seconds with negligible error, which makes real-time accurate flow reconstruction possible. They obtained flow velocities of much higher magnitudes compared to the results reported in the previous literature, which revealed that the previous results were underestimated. This method shows the potential of deploying neural network models to achieve real-time flow field reconstruction and measurement.

#### 3.2.2. Flow Velocity Reconstruction by Physics-Informed Neural Networks

A physics-informed neural network (PINN) is a variant of neural networks that embeds prior knowledge of physical laws and equations governing certain physical processes into model training. Therefore, even with a low amount of training data, an optimized solution aligning the underlying physical mechanism can be found.

Recently, Wang et al. [38] leveraged PINNs to approximate velocity and pressure fields in high resolution. The prior knowledge on the Reynolds number and the Navier–Stokes equations serves as the regularization term in the loss function. The dataset included velocity and pressure data from experimental measurement and analytical solutions, DNS data, and particle images obtained by a time-resolved tomographic PIV/PTV. A simulation on 2D Taylor’s decaying vortices is used to test layer sizes, activation functions, and optimizers. A numerical wall-bounded turbulent flow at $Re_{\tau }$ = 550 was applied to evaluate the overall performance of the proposed PINN. Velocity reconstructions by the PINN on sparse experimental data outperform those by the tomographic PIV/PTV in a 3D wake flow of a hemisphere but miss the small-scale vortical structures in the DNS data. Pressure fields can be reconstructed with larger error (about one order bigger of magnitude) compared to the error in velocity fields.

However, reconstructing inhomogeneous and anisotropic fields is still challenging for the PINN at the current stage. The relatively low converging speed is another important issue. Due to the noise brought by spatial cross-correlation in the PIV algorithm, the authors conclude that PINNs can achieve better performance with data of high quality and resolution obtained from PTV instead of PIV.

### 3.3. Deep Neural Networks-Assisted Particle Tracking

Deep neural networks (DNNs), particularly the convolutional neural networks (CNNs), have become the popular mainstream techniques when it comes to image processing and computer vision in deep learning.

Models built upon DNNs or CNNs typically consist of dozens to hundreds of hidden layers to perform highly complicated linear and nonlinear transformations. Although they need significantly larger memory storage and exhibit substantially more complex structures compared to SNNs, they also offer unparalleled performance in tasks such as image classification, object detection, and segmentation. One of the key reasons behind their success is their ability to automatically learn hierarchical representations of data, starting from low-level features like edges and textures and gradually progressing to more abstract and high-level concepts. This hierarchical feature learning enables CNNs to effectively capture intricate patterns and variations within images, making them robust to changes in lighting, viewpoint, and occlusion.

#### 3.3.1. Long Short-Term Memory-Based Recurrent Prediction on Particle Locations

**Figure 6:**
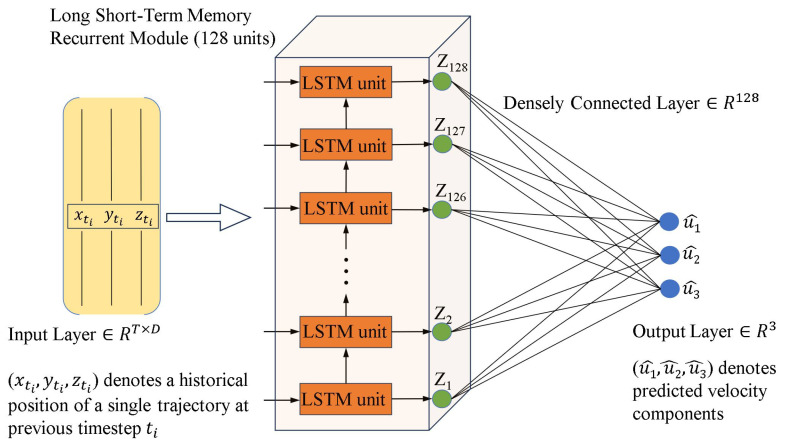
The procedure for the proposed LSTM recurrent predictor to predict the velocity components of a single particle based on a temporal sequence of previous location data (input), adapted from Mallery et al. [39].

Mallery et al. [39] introduced a data-driven method for dense particle tracking using a learning predictive model, aiming to address the limitation of particle velocity resolution encountered by traditional methods. They proposed a model (Figure 6) based on a Long Short-Term Memory (LSTM) recurrent neural network [97], which can accurately predict the future velocity of particles based on their past positions. Ground truth data for model training were obtained either through manual operation with a conventional tracking tool to record meaningful trajectories or by running a supplemental easy-tracking experiment. Experimental validation shows that this method has superior performance, by increasing the total number of links by approximately 30% compared to prior multi-pass (iterative) tracking approaches, with a manual assessment revealing only 1% of these links to be incorrect. The proposed LSTM method improves the quality and accuracy in tracking particles, especially under high particle concentration or complex flow conditions. The method also demonstrates potential value for non-fluid measurement applications, such as studying the swimming behavior of micro-algae in biological research.

#### 3.3.2. DeepPTV: A Deep Neural Network-Based Framework

**Figure 7:**
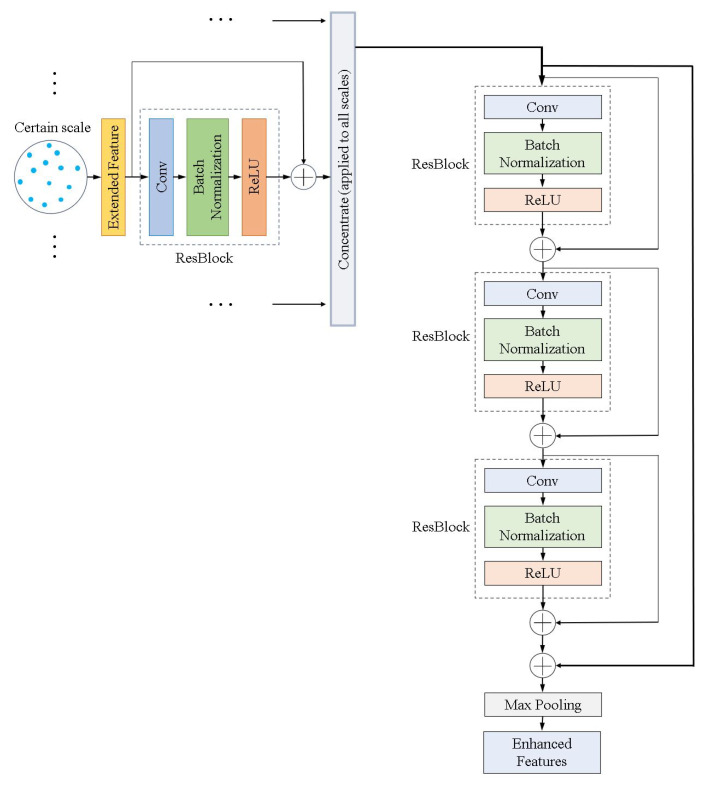
Structure of the proposed enhanced multiscale feature learner using multiple ResBlocks to extract features from different local spatial scales in DeepPTV. The internal structure of each ResBlock is shown in the small dashed box. The output enhanced feature information could be the integration of particle’s size, location, and the relative distances between it and its neighboring particles, etc. Adapted from Liang et al. [37].

Liang et al. [37] proposed a novel architecture called DeepPTV, which uses deep neural networks to learn complex fluid motion across different scales (Figure 7). The DeepPTV architecture features an enhanced multi-scale feature learner and a convection architecture. The enhanced multi-scale feature learning layer integrates local spatial geometry information with more robust features to reinforce the generalizability for complex nonrigid flow motion. This layer combines features from local spatial structures of multiple scales, addressing challenges posed by various particle densities and flow motion magnitudes across different local regions. It aims to strike a balance between accuracy and robustness in motion estimation by extracting multi-scale extended features from several local circular regions with different radii. The convection architecture approximates complex nonrigid flows using a hierarchical approach from large scale to small scale, enhancing the extraction of large-scale motion fields and the refinement of small-scale structures. It involves stacking multiple networks to form a “convection” for flow refinement, which is the first application of the stacking scheme to flow estimation on point clouds. Synthetic and public datasets on particle locations and flow fields were obtained for model training. The results shows that DeepPTV can achieve high accuracy and robustness on synthetic datasets. In evaluations under different parameter conditions, DeepPTV also demonstrated superior performance, particularly in handling small-scale flow structures.

Compared to some other data-driven tracking methods, e.g., FlowNet3D [98], DeepPTV exhibits better robustness in dealing with off-plane motion and particle position noise, with higher computational efficiency. However, only two subnetworks are placed by the authors in the presented framework. The accuracy can be further improved with more subnetworks added to enhance the generalizabilty of the model; but the refinement also incurs additional computational time and resource utilization.

#### 3.3.3. A Hybrid Convolutional Deep Neural Network Architecture to Estimate 3D Position and Size of Particles

**Figure 8:**
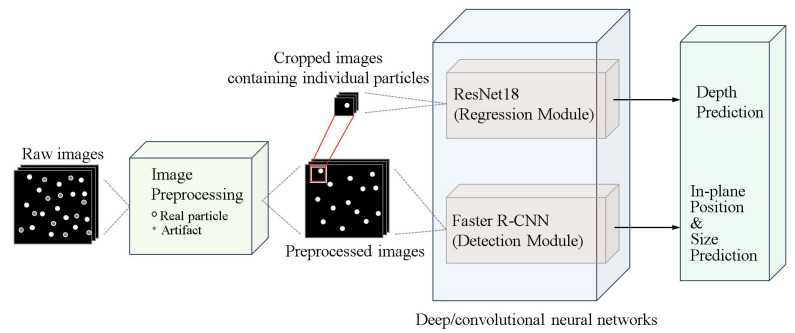
Basic pipeline of the proposed deep hybrid dual neural networks framework using ResNet18 (for depth regression) and Faster R-CNN (for in-plane position and particle size prediction). The image preprocessing includes filtering and labeling. Adapted from Ratz et al. [99].

Recently, Ratz et al. [99] used a hybrid convolutional deep neural network framework with ResNet18 [100] and Faster R-CNN [84] to reliably determine the 3D position and size of particles from a single camera view (Figure 8). Synthetic and experimental datasets were used to train and evaluate the model. They showed that the performance of this method on real images is similar to that on synthetic images, showing similar trends. In real images, the uncertainty of the plane in all cases is less than 0.5 pixels, and the uncertainty of the depth position is about 1 micron, even in the presence of multiple distributions of particles of different sizes. In addition, the method exhibits excellent classification performance with a minimum accuracy above 96%. This study demonstrates that these CNN models primarily targeted on image classification tasks have great potential in the locating of particles in 3D volume and can be applied in the practical field of microfluidics, providing higher accuracy and reliability for micro-scale volumetric measurements.

### 3.4. Transformer and Attention Mechanism-Assisted Particle Tracking

Transformers represent a paradigm shift in the field of deep learning, particularly in natural language processing (NLP) and sequence modeling tasks. Unlike traditional DNNs and CNNs, which rely on sequential processing and hierarchical feature extraction, transformers employ a self-attention mechanism [101] to capture long-range dependencies within sequences more efficiently.

The attention mechanism allows transformers to consider all input tokens simultaneously, enabling parallelization and reducing the computational complexity of modeling sequential data. As a result, transformers have demonstrated remarkable success in various NLP tasks, including machine translation, text generation, and sentiment analysis, often outperforming previous approaches based on recurrent neural networks (RNNs) and CNNs. Furthermore, transformers have shown versatility beyond NLP, being applied to image processing, time-series forecasting, and other domains, showcasing their potential to reshape the landscape of deep learning architectures and techniques. There are comparatively few studies on the application of transformer models on particle tracking and flow reconstruction [94,95,102,103,104]. Most of them focus heavily on flow estimation rather than individual particle detection and tracing, which is out of the scope of this article.

### 3.5. Key Takeaways from Data-Driven Methods

Although data-driven methods are just introduced to this domain and still evolving, they have demonstrated substantial potential in particle tracking and flow field reconstruction (see Table 2). These data-driven algorithms can directly learn the complex relationships between input data and particle trajectories or velocity fields, with enhanced accuracy and resolution. By training on large datasets of experimental or synthetic flow data, data-driven models can capture intricate flow dynamics and variability across multiple scales that may be challenging to represent using classical analytical or numerical approaches.

Future improvements may focus on expanding working scales, improving converging rates, inventing end-to-end frameworks, and also advancing current experimental setups. Architectures should be optimized for real-time processing, robustness to noise and occlusions, and adaptability to diverse experimental conditions. Efforts are needed to integrate physics-based regularization and domain knowledge into data-driven models, such as incorporating fluid dynamics principles into neural network training, which could lead to more interpretable and physically meaningful results.

The development of hybrid approaches that combine the strengths of data-driven techniques with traditional numerical simulations or analytical methods could offer complementary insights and validation mechanisms, enhancing the overall reliability and confidence in particle tracking.

## 4. Conclusions

This article presents a summary of advanced micro-scale particle tracking techniques, including conventional methodologies and emerging data-driven approaches. The conventional methods discussed, such as tomographic PTV, ’Shake-The-Box’, confocal microscopy, and plenoptic imaging, have long served as reliable tools for capturing particle motion at the micro-scale level.

Limitations of traditional techniques, including limited versatility and adaptability across flow scenarios, restricted spatial and temporal resolutions (especially under high seeding densities), demanding computational load, distracting image artifacts, and background noises, point to the need for continual advancements. The integration of data-driven methods, such as deep learning algorithms and state-of-the-art computer vision techniques, presents a paradigm shift in micro-scale particle tracking. These innovative approaches offer the potential to overcome inherent challenges by enhancing accuracy, efficiency, robustness, and generalizability. The synergy of traditional and data-driven methods, as highlighted in this review, promises for a more holistic and robust understanding of micro-scale particle dynamics, opening avenues for breakthroughs in fields ranging from fluid mechanics to biophysics. These advanced methodologies are critically needed to track particles in challenging environments and complex geometries. Synergistic methods combining tracking with computational fluid dynamics can validate and refine numerical models, improving the accuracy of predictions for engineering design and optimization tasks in aerospace, automotive, and energy sectors. In medical diagnostics, high-precision particle tracking can enhance the detection and monitoring of diseases by tracking the motion of biomarkers, pathogens, and therapeutic agents in biological fluids such as blood circulation or tissue microenvironments. Synergistic methods combining tracking with imaging modalities like fluorescence microscopy or magnetic resonance imaging (MRI) can provide spatial and temporal information aiding in the development of personalized diagnostics and targeted therapies. As research in these interdisciplinary fields advances, the combination of traditional and data-driven methods is prepared to push micro-scale particle tracking into new areas. This will deepen our understanding of fluid systems and enable transformative applications across various scientific and technological fields.

## Figures and Tables

**Table 1:** A summary of four noteworthy 3D PTV methods.

Approach	Features	Advantages	Disadvantages	Literature
(Digital) Holographic	Records the interference pattern of light scattered by objects (hologram)	Single camera; suitable for limited space experiments	Slower processing speed compared to tomographic methods; low particle density; additional light source required	[45,46,47,48]
Confocal	Confocal microscopy performs fast scanning across various depth planes	Single camera; high resolution; high particle density	Slow capture speed limits ability to track slow-moving particles; specialized microscopy equipment required	[22,23,24,25]
Tomographic	Multiple cameras capture images of tracer particles from different angles	High resolution; high particle density; time-resolved tracking	Complex calibration and limited flexibility in micro-scale settings due to space constraints; high equipment cost (multiple cameras and synchronizers)	[4,5,6,7,8]
Light Field (Plenoptic)	Allows post-capture synthetic refocusing; uses micro-lens arrays (MLAs)	Single camera; pre-calibrated; high processing speed	Low particle density; intense illumination needed; varying micro-lens arrays (MLAs) required for different particle sizes; high computational cost for 3D reconstruction	[33,34,35,64,65,66]

**Table 2:** A summary of representative data-driven methods.

Approach	Features	Advantages	Disadvantages	Literature
Two-layer SNN	Obtains the mapping function for 3D stereoscopic PTV	Simple model structure; low computational load for learning mapping functions; high accuracy in reconstructing particle positions	Limited versatility due to shallow structure; low seeding density of particles (0.004 ppp)	[36]
PINN	Incorporates the governing physics as a regularization technique	Suitable for low data volumes; can simultaneously reconstruct other properties (e.g., pressure fields) with velocity fields	Limited versatility in inhomogeneous and anisotropic fields; slow convergence rate; long training period; limited accuracy	[38]
SPAV	Uses a statistical loss that considers arbitrary localization and tracking uncertainties; implemented with a PINN	High versatility; suitable for all forms of PTV; increased accuracy and robustness compared to conventional PINNs	Loss components need refining to consider isotropic errors; inconsistency in non-Gaussian advected particle probability density functions under strong velocity gradients; limited computational efficiency and domain size in PINN SPAV (resolving finer scales of intense turbulent flows is limited)	[40]
LSTM	Incorporates past particle locations to refine predictions of future particle velocities	Strong robustness under high seeding densities and complex flow conditions	Requires additional input information to improve performance; increased input dimensionality	[39]
DeepPTV	Deploys an enhanced multi-scale feature learner and a convection architecture	Applicable for tracking simultaneous multi-scale flow motions	Limited to two subnetworks; accuracy could improve with more subnetworks but would require more computational resources	[37]
Hybrid DNN	Combines convolutional deep neural networks (ResNet18 + Faster R-CNN) for depth regression and particle classification	Single camera view; high versatility for particles of different sizes; high classification accuracy	Decreased performance (recall) under extremely high seeding densities; higher uncertainty in regression and classification of similar particles at close proximity	[99]
